# Supplementary figures and images for: Genome-wide identification and expression analysis of the phosphatase 2A family in rubber tree (Hevea brasiliensis)
Source: PLoS One. 2020 Feb 5;15(2):e0228219. doi: 10.1371/journal.pone.0228219 (PMC7001923; doi:10.1371/journal.pone.0228219)

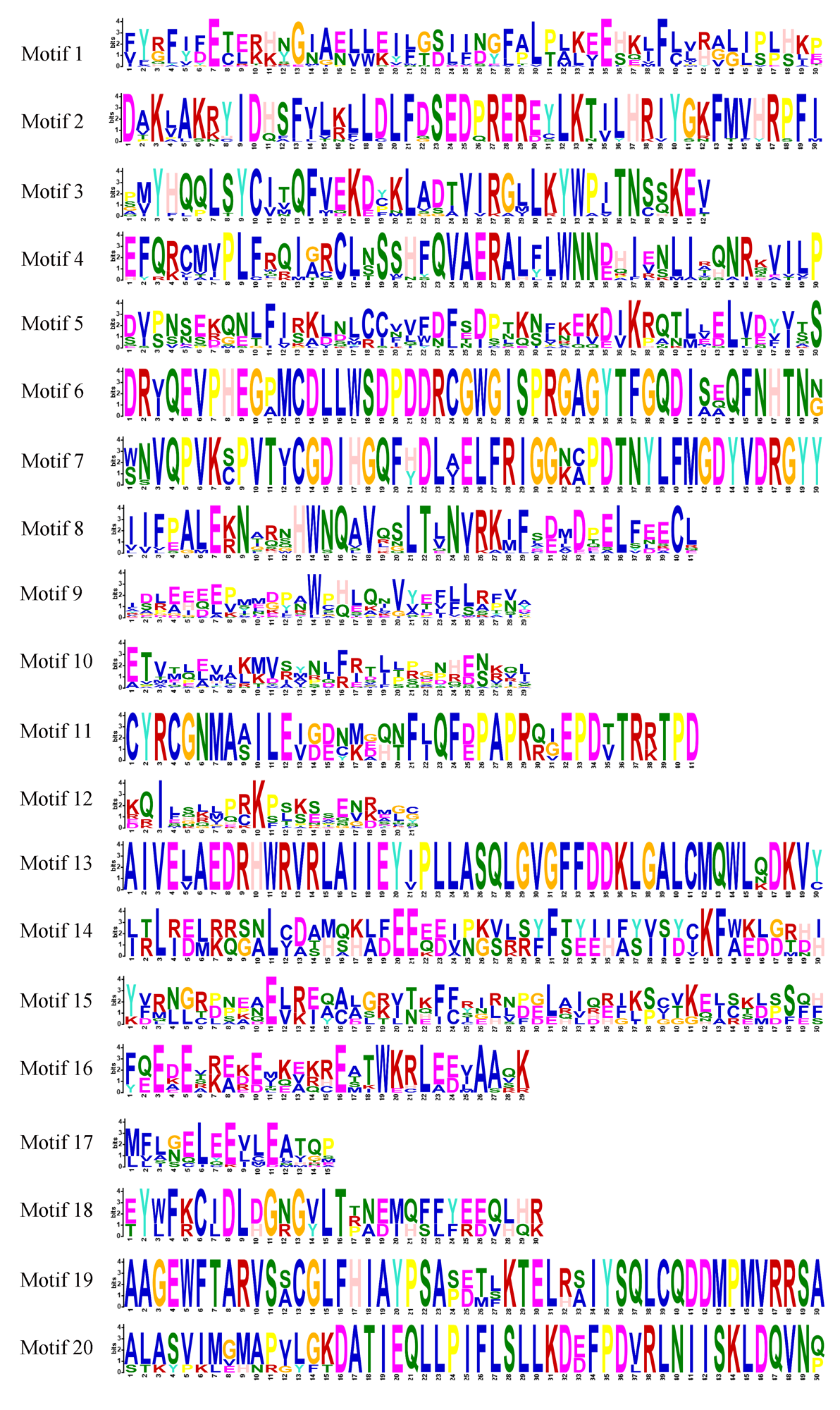

Supplement: S1 Fig — (TIF) [file pone.0228219.s001.tif]
