# Supplementary material for: Genome-wide identification and expression analysis of the phosphatase 2A family in rubber tree (Hevea brasiliensis)
Source: PLoS One. 2020 Feb 5;15(2):e0228219. doi: 10.1371/journal.pone.0228219 (PMC7001923; doi:10.1371/journal.pone.0228219)
Supplement: S1 Table — (DOCX) [file pone.0228219.s002.docx]

**S1 Table. Primers used in this paper.**

| Primers Name | Primer sequence |
| --- | --- |
| *HbPP2AA1-1-qF* | CAAGCACTACAGTC |
| *HbPP2AA1-1-qR* | GTGGAAGAAACCTC |
| *HbPP2AA1-2-qF* | TCTTCTTGCTCCTGTTAT |
| *HbPP2AA1-2-qR* | ACGAATTGTCTTCTCCAC |
| *HbPP2AA3-qF* | CCCAGATGAAGGAGAGTG |
| *HbPP2AA3-qR* | TTTTAACATATCAGAGGC |
| *HbPP2AB'α-qF* | AAGAGCAGCGGGAAAT |
| *HbPP2AB'α-qR* | CCAAAATGGGGAGGAG |
| *HbPP2AB'β-qF* | GAGCAACGGGAAATGA |
| *HbPP2AB'β-qR* | TAGCACCAAAATGGGG |
| *HbPP2AB'γ-qF* | TTGAATGGTAGAGGGGAC |
| *HbPP2AB'γ-qR* | AGAGAAAATAAAGCAGAA |
| *HbPP2AB'ζ-qF* | AAGCTGTTCAGAGTCTAA |
| *HbPP2AB'ζ-qR* | CATCCTCCAATCTTTTCC |
| *HbPP2AB'η-1-qF* | CTGCCCATAATCTTCCCT |
| *HbPP2AB'η-1-qR* | CAGCCTTTGCTTCCTCTT |
| *HbPP2AB'η-2-qF* | CAAGAAGATGAAGCAAGG |
| *HbPP2AB'η-2-qR* | CTGAAGATGTTAGGGTGG |
| *HbPP2AB'η-3-qF* | TTGGTTATCCTGGAGG |
| *HbPP2AB'η-3-qR* | CATGGGCAGACTTTTC |
| *HbPP2AB'η-4-qF* | GGCTAGATAGTTGGAA |
| *HbPP2AB'η-4-qR* | AAGAAGGTTTAGGTGA |
| *HbPP2AB'θ-1-qF* | ACATAAGTTTGAAGAAGC |
| *HbPP2AB'θ-1-qR* | CTAACCTGAAGAGGAGTG |
| *HbPP2AB'κ-1-qF* | AGTGAAGAAAAACTAAGC |
| *HbPP2AB'κ-1-qR* | AGAAAATAACAACAAGAA |
| *HbPP2AB''α-qF* | ATGGAAGAAGATGTAGAG |
| *HbPP2AB''α-qR* | AGTAGCATAAATTGATGA |
| *HbPP2AB''β-qF* | CAGGTGACAGAAGTTC |
| *HbPP2AB''β-qR* | ATACACGGATGAGGAT |
| *HbPP2AB''δ-qF* | GACCAGAGAACGAGAGCT |
| *HbPP2AB''δ-qR* | TCCTGACGAATCAGAAAT |
| *HbPP2AB''ε-qF* | TAGCACCAAAATGGGG |
| *HbPP2AB''ε-qR* | CAGCACCCAAGTTGTT |
| *HbPP2A-TON2/FASS1-1-qF* | TGCTAACACTGAACAG |
| *HbPP2A-TON2/FASS1-1-qR* | CAACTTACAAACAACT |
| *HbPP2A-TON2/FASS1-2-qF* | GTAAACAGGGAGGGAC |
| *HbPP2A-TON2/FASS1-2-qR* | AAACATGCAACGAAAC |
| *HbPP2AB55α/Bα-1-F* | TGGGAAGATCATGGAC |
| *HbPP2AB55α/Bα-1-R* | ATATAATTAGCGGGGC |
| *HbPP2AB55β/Bβ-F* | TGTTCCGCGTGTTTGGTT |
| *HbPP2AB55β/Bβ-R* | ACTCCTGGGCTTTCCGTT |
| *HbPP2AC1-1-F* | TTGGGGTTTACCTGAT |
| *HbPP2AC1-1-R* | TTATTGCTGTTCCTTC |
| *HbPP2AC1-2-F* | GCTCACCAGCTTGTTATG |
| *HbPP2AC1-2-R* | GAGGAAATTCTGCTCCAT |
| *HbPP2AC2-1-F* | AGAACATGGACCAGAACT |
| *HbPP2AC2-1-R* | CACCTTAAACAACAAAAA |
| *HbPP2AC2-2-F* | AATTTCCATATTTCTG |
| *HbPP2AC2-2-R* | GGTTTTACTTGAGCAC |
| *HbPP2AC4-1-F* | TGGTTATTGCCTCCTA |
| *HbPP2AC4-1-R* | CTCCACTGCTTCCTCT |
| *HbPP2AC4-2-F* | TTGTTATCGCTGTGGAAA |
| *HbPP2AC4-2-R* | AGCTAGGAGGCACTAGTC |
| *HbPP2AC4-3-F* | GAAACCTCGCTGGATATG |
| *HbPP2AC4-3-R* | GGTGAAAAAAGAGTAAAA |
| *HbPP2AC6-F* | CTGCCCCTTCTCCCTA |
| *HbPP2AC6-R* | CACAAAATCTTGCCCT |
| *HbUBC2b-F* | CGACCAAGTTTTCATTTCGGGTG |
| *HbUBC2b-R* | AGTCTCTTCTTTGCTGGGGTTG |
