# Supplementary material for: Genome-wide identification and expression analysis of the phosphatase 2A family in rubber tree (Hevea brasiliensis)
Source: PLoS One. 2020 Feb 5;15(2):e0228219. doi: 10.1371/journal.pone.0228219 (PMC7001923; doi:10.1371/journal.pone.0228219)
Supplement: S2 Table — (DOCX) [file pone.0228219.s003.docx]

**S2 Table. Protein sequences used in this paper.**

>HbPP2AA1-1

MATIDEPLYPIAVLIDELKNEDIQLRLNSIRKLSTIARALGEERTRKELIPFLSENNDDDDEVLIAMAEELGVFIPYIGGVEHANVLLPPLETLCTVEETCVRDKAVESLCRIGVQIREQDLVEYFIPLVKRLAAGEWFTARVSSCGLFHIAYPSAPETLKTELRAIYSQLCQDDMPMVRRSAATNLGKFAATIEPAHLKTDIMSIFEDLTQDDQDSVRLLAVEGCAALGKLLEPQDCVAHILPVIVNFSQDKSWRVRYMVANQLYELCEAVGPELTSSDLVPAYVRLLCDNEAEVRIAAAGKVTKFCRILNPELAIQRIIPCVKELSTDSSQHVRSALASVIMGMAPILGKDATIEQLLPIFLSLLKDEFPDVRLNIISKLDQVNQVIGIDLLSQSLLPAIVELAEDRHWRVRLAIIEYVPLLASQLGVGFFDDKLGALCMQWLKDKVYSIRDAAANNVKRLAEEFGPDWAMQHIVPQVLDMINNPHYLYRMTILHAISLLAPVMGSEITCSTLLPVVVNTSKDRVPNIKFNVAKVLQSLIPIVDQSVVDKTIRPCLVELSEDPDVDVRFFATQALQSSDQVMMST*

>HbPP2AA1-2

MATIDEPLYPIAVLIDELKNEDIQLRLNSIRRLSTIARALGEERTRKELIPFLSENNDDDDEVLLAMAEELGVFIPYVGGVEHANVLLPPLETLCTVEETCVRDKAVESLSRIGAQMREQDLVEYFIPLVKRLAAGEWFTARVSSCGLFHIAYPSASETFKTELRAIYSQLCQDDMPMVRRSAATNLGKFAATVEPAYLKTDIMTIFEDLTQDDQDSVRLLAVEGCAALGKLLEPQDCVAHILPVIVNFSQVWHAFIFVSVHFLVVIYFFNVGIRADLVPAYVRLLCDNEAEVRIAAAGKVTKFCRILNPELAIQRILPCVKELSTDSSQHVRSALASVIMGMAPVLGKDATIEQLLPIFLSLLKDEFPDVRLNIISKLDQVNQVIGIDLLSQSLLPAIVELAEDRHWRVRLAIIEYIPLLASQLGVGFFDDKLGALCMQWLNDKVYSIRDAAANNVKRLAEEFGPDWAMQHIVPQVLDMINNPHYLYRMTILHAISLLAPVMGSEITCSTLLPVVVSASKDRVPNIKFNVAKVLQSLIPIVDQSVVEKTIRPCLVEMSEDPDVDVRFFATQALQSSDQVMMST*

>HbPP2AA2

MSMVDEPLYPIAVLIDELKNDDIQLRLNSIRRLSTIARALGEERTRKELIPFLSENNDDDDEVLLAMAEELGVFIPYVGGVEHAHVLLPPLETLCTVEETCVRDKAVESLCRIGSQMREGDLVDWFIPLVKRLAAGEWFTARVSACGLFHIAYPSAPDTSKTELRSIYSQLCQDDMPMVRRSAASNLGKFAATVEPAHLKIDIMSIFEDLTQDDQDSVRLLAVEGCAALGKLLEPQDCVAHILPVIVNFSQDKSWRVRYMVANQLYELCEAVGPEPTRTDLVPAYVRLLRDNEAEVRIAAAGKVTKFCRILNPELAIQHILPCVKELSSDSSQHVRSALASVIMGMAPVLGKDATIEQLLPIFLSLLKDEFPDVRLNIISKLDQVNQVIGIDLLSQSLLPAIVELAEDRHWRVRLAIIEYIPLLASQLGVGFFDDKLGALCMQWLQDKVYSIRDAAANNLKRLAEEFGPEWAMQHIIPQVLEMINNPHYLYRMTILRAVSLLAPVMGSEITCSKLLPVVINASKDRVPNIKFNVAKVLQSLIPIVDQSVVEKTIRPCLVELSEDPDVDVRFFANQALQAIDNVMMSS*

>HbPP2AA3

MSVVDEPLYPIAVLIDELKNDDIQLRLKTIRRLSTIARALGEERTRKELIPLLSENNDDDDEVLLTMAEELGVFIPYVGGVEHAHVLLPPLETLCTVEETCVRDKAVESLCRIGSQMKESDLVDWFIPLVKRLAAGEWFTARVSACGLFHIAYPSASDMLKTELHSIYSQLCQDDMPMVRRSAASNLGKFAATVEPAHLKTDIMSIFEDLTQDDQDSVRLLAVEGCAALGKLLEPQDCIAHILPVIVNFSQDKSWRVRHMVANQLCKLCEAMGPEPTRTDLVPAYVRLLRDNEAEVRIAAAGKVTKFCCILNPELAIQHILPCVKELSSDSSQHVRSALASVIMEMAPVLGKDATIEQLLPIFLSLLKDDFPDLRLNIISKLDQVNPVIGIDLLSQSLLPAIVELAEDRHWRVRLAIIEYIPLLASQLGVGFFDDKLGALCMQWLQDKVCVILSCATEVIKYNSLLSPTLVLH*

>HbPP2AB'α

MLKKIIKGGHKKPSKSDGNDSGFGPPGTRNSGSGPASNVVVNHASRTTPGPVTPNNGTTVVAPPPMNSVEPLPLFRDVPVSERQNLFLRKLQVCCFQLDFSDTLKSVREKEIKRQTLLELVNFIQSGSGKITETCQEEMIRMVSVNIFRCLPPASHENTGQEVADPEEEEPYLEPSWPHLQLVYELLLRYVVSSDTDTKVAKKYIDHSFVLKLLDLFDSEDPREREYLKTILHRIYGKFMVHRPFIRKAINNIFYRFIYETERHSGIGELLEILGSIVNGFALPMKEEHKLFLVRALIPLHKPKPISVYHQQLSYCIVQFVEKDYKLADTVIRGLLKYWPLTNCQKEVLFLGELEEVLEATQSAEFQRCMVPLFRQIARCLTSSHFQVAERALFLWNNEHIVSLIAQNRSVILPIIFEALEKNIQSHWNQAVHGLTVNVRKMFLEMDTELFEECQRQYEEKESRAKEVEEQREMTWKRLADVAAQREGDDMVSV*

>HbPP2AB'β

MLKKIMKGGHKKPSKSDGNDYGVAPLGTRNSGSGPASNVVVNHASRTAPSPPNNGTTVAAPPPMSSVETLPQFRDVPVSERQNLFLRKLQVCCFQLDFSDPLKSVREKEIKRQTLLELVDFIQSGSGKITETCQEEMIRMVSVNVFRCLPPASHENTGQEAADPEEEEPYLEPSWPHLQLVYELLLRYVVSSDTDTKVAKRYIDHSFVLKLLDLFDSEDPREREYLKTILHRIYGKFMVHRPFIRKAINNIFYRFVYETERHSGIGELLEILGSIINGFALPMKEEHKLFLVRALIPLHKPKPISVYHQQLSYCIVQFVEKDYKLADTVIRGLLKYWPLTNCQKETDCSLPHFLFLGELEEVLEATQSAEFQRCMVPLFRQIARCLTSSHFQVAERALFLWNNEHIVSLIAQNRTVILPIIFEALEKNIQSHWNQAVHGLTVNVRKMFLEMDTELFEECERRYQEKESRAKEVEEQREMTWKRLADVAAQKVGEDMVTV*

>HbPP2AB'γ

MIKQILGKLPRKPSKSSNNDSNNDGGVNAFSSLNSSHGPNSINSSKASSIASKSASSGLGASRVNNGTIAPQNKSNQGKKSAAVAGQVGPMLASGVYEPLPNFKDVPSSEKQNLFIRKLNMCCVVFDFSDPSKNLKEKDIKRQTLHELVDYISSVTSKFNEVTMQEITKMAAANLFRTLPSVNHDNKILEMYDPEEDEPTMEPAWPHLQIVYEFLLRFVASTETDAKLAKRYIDHSFVLKLLDLFGSEDQREREYLKTILHRIYGKFMVHRPFIRKSINNIFYRFIFETERHNGIAELLEILGSIINGFALPLKEEHKLFLVRALIPLHKPKCVSMYHQQLSYCITQFVEKDFKLADTVIRGLLKYWAITNSSKEVMFLGELEEVLEATQAAEFQRCMIPLFRQIGRCLNSSHFQVAERALFLWNNDHIRNLITQNRQVILPIIFPALERNTRGHWNQAVQSLTLNVRKIFSDADQELFDDCLVKFQEDEVKEREMQEKRESIWKRLEDVAASKAVSNEAVLVSRFASAVAITAGTGPCATAGS*

>HbPP2AB'ζ

MIKQILGKLPRKPSKSSNNDSNNDGGVNAFSSLNSSHGPNSINTSKSSSISSKSNSGLGASRMNNGTIAPQNKSNQGKKSATVAGQVGPMLASGVYESLPNFRDVPSSERQSLFIRKLNMCCVVFDFSDPSKNLKEKDVKRQTLLELVDYISSVTSKFNEVTMQEITKMVAANLFRSLPSSNHDNKILETYDPEEEEPTMEPAWPHLQIVYEFLLRFVASTETDAKLAKRYIDHSFVLKLLDLFDSEDQREREYLKTILHRIYGKFMVHRPFIRKAINNIFYRFIFETERHNGIAELLEILGSIINGFALPLKEEHKLFLVRALIPLHKPKCVPMYHQQLSYCITQFVEKDFKLADTVIRGLLKYWPITNSSKEVMFLGELEEVLEATQPAEFQRCMIPLFRQIGRCLNSSHFQVAERALFLWNNDHIRNLITQNRNVILPVIFPALERNTRGHWNQAVQSLTLNVRKIFSDADQELFDECLVKFQEDEVKEREIQERRESTWKRLEDVAASKAISNEAVLVSRFASSVAITTVTSPHSTAGS*

>HbPP2AB'η-1

MIKQILGRLPRKPSKSSENREFGGPSAPSNTSITSRSSDLVSNRPVTLNNASPPASDSASYLGYGHGSKPTQALSPKLNGNSVISPYEPLPGFKDVPNSEKQNLFIRKLKLCCVVFDFNDPTKNLKEKDIKRQTLVELVDYVTSANWKFSETVIEESIKMVSVNLFRSLTPQPRENKVLEAFDLEEEEPLMDPAWPHLQIVYEFFLRFVASPETDAKLAKRYIDHSFVLKLLDLFDSEDPREREYLKTILHRIYGKFMVHRPFIRKAINNIFFRFIFETEKHNGIAELLEVLGSIINGFALPLKEEHKLFLVRALIPLHKPKCLPMYHQQLSYCITQFVEKDCKLADTVIRGLLKYWPITNSSKEVMFLSELEEILEATQPAEFQRCMVPLFHQIARCLSSSHFQVAERALYLWNNDHIENLIRHNRKIILPIIFPALEKNGRNHWNQLVQSLTVNVRKIFADIDPELFEECLKQFEEEEAKADDLKAKHEATWKCLEEIAAKKAASSEAVHVPHTVPTLTAHNLPCLGKEWT*

>HbPP2AB'η-2

MIKQILGRLPRKPSKSSENREFSGQSAPSSNTSTGSKSSNLVSNKPSTLNNSSTLVSDSASHLGYGHGTKPTQSVNQKLNDNSVVAPYEALPGFKDVPNSEKQNLFIGKLNLCCVVFDFSDPTKNLKEKDIKRQTLVELVDYIASANGKFTETVVQEVIKMVSMNLFRSLTPQPRENKVLEAFDLEEEEPLIDPAWPHLQIVYEFFMRFVASPETDAKLAKRYIDHSLVLKLLDLFDSEDPREREYLKTILHRIYGKFMVHRPFIRKAINNIFFRFIFETEKHNGIAELLEVLGSIINGFALPLKEEHKLFLVRALIPLHKPRCLPMYHQQLSYCITQFVEKDCKLVDTVIRALLKYWPITNSSKEVMFLSELEEVLEATQPAEFQRCVVPLFRQIARCLSSSHFQVAERALYLWNNDHIENLIRQNRKVILPIIFPALEKNGSNHWNQVVQSLTVNVRKIFADVDPELFEECLKQFQEDEARAEDMKVKHEATWKHLEEIAARKAAGSEAVLVPHAIPTLTSSG*

>HbPP2AB'η-3

MIKQILNRLPRKPSKSSENREGGGTSTSSSNASTSPRNGDLSGNRYASAGATSVAGFNPTSNLGTNYGNKLSQAVNAKLNGNQPAPYEPLPSFRDVPNSEKQNLFIRKLNLCCIVFDFTDPTKNFKEKDIKRQTLVELVDYVSSANGKFPETMMQEMIKMVSSNLFRILSSPPRENKILEAYDLEEDEPSMDPAWPHLQVVYEFLLRFIASPETDAKLAKRYIDHSFVLRLLDLFDSEDPREREYLKTVLHRIYGKFMVHRPFIRKAINNIFYRFIFETEKHNGIAELLEILGSIINGFALPLKEEHKLFLVRVLIPLHKPKCLPMYHQQLSYCITQFVEKDCKLADTVIRGMLKYWPITNSSKEVMFLGELEEVLEATQPAEFQRCMVPLFRQIGRCLSSSHFQVAERALFLWNNDHIENLIQQNRKVILPIIFPALERNARQHWNQAVQSLTLNVRKIFSDIDLELFEECLLKFQEDETREKEMKVKRETTWKRLEEMAAVKATSNEPVLVSPKMMTRALSG*

>HbPP2AB'η-4

MIKQIFNRFPRKPSKSSENREGGGTSTSSSNAFSSSRNNDLSGNRYASQGATSLAGFNSTSNLGSNYGNKLSQVVNSKLNGSQPPPYEALPSFRDVPNSEKQNLFIRKLNLCCFVFDFSDPTKNFKEKDIKRQTLVELVDYVSSANGKFSETMMQEMIKMVSINLFRTLSSPPRENKVLEAFDLEEDEPSMDPAWPHLQVVYEFLLRFVASPETDAKLAKRYIDHSFVLRLLDLFDSEDPREREYLKTVLHRIYGKFMVHRPFIRKAINNIFYRLIFETEKHNGIAELLEILGSIINGFALPLKEEHKLFLVRALIPLHKPKCLPMYHQQLSYCITQFVEKDCKLADTVIRGMLKYWPITNSSKEVMFLGELEEVLEVTQPAEFQRCMVPLFRQIGRCLNSSHFQVAERALFLWNNDHIENLIRQNRKVTLPIIFPALERNARQHWNQAVQSLTLNIRKIFSDIDPELFEECLVKFQEDETREKETKVKRETTWKRLEEIAAVKAASNEPVLVSPKIMPRAPSG*

>HbPP2AB'η-5

MIKQIFNRFPRKPSKSSENREGGGTSTSSSNAFSSSRNNDLSGNRYASQGATSLAGFNSTSNLGSNYGNKLSQVVNSKLNGSQPPPYEALPSFRDVPNSEKQNLFIRKLNLCCFVFDFSDPTKNFKEKDIKRQTLVELVDYVSSANGKFSETMMQEMIKMVSINLFRTLSSPPRENKVLEAFDLEEDEPSMDPAWPHLQVVYEFLLRFVASPETDAKLAKRYIDHSFVLRLLDLFDSEDPREREYLKTVLHRIYGKFMVHRPFIRKAINNIFYRLIFETEKHNGIAELLEILGSIINGFALPLKEEHKLFLVRALIPLHKPKCLPMYHQQLSYCITQFVEKDCKLADTVIRVASLVLICWCYLSFA*

>HbPP2AB'θ-1

MLKQILSKLPRKSYKSTENRDRGRNLATNSYNSTGSRSCDLGAKSAKLNITSVTAYDSASDRGQNHGNDIQQGVTSKLNGILGFSSYEGLPELRDVPNSEKQNLFIKKLNLCCIVFDFSDPTKNLKEKDIKRQTLLELVDYVTSVNGKFTETVMQEVIKMVSANLFRTISPQPHENKVVDGVDLEEEEPSMDLAWPHLQLVYELFLRFVASSETDVKPAKRYIDQSFILKFLDLFDSEDPREREYLKTILHRIYGKFMVHRPFIRKAINNIFYQFIFETEKHNGIAELLEILGSIINGFALPLKEEHKMFLIHALIPLHKPRCLAMYHQPLSYCIMQFVEKDYKLADAVIRGLLKYWPLTNSSKEVMFLNELEEVLEATQPSEFQRCTVPLFQKIAQCLNSSHFQVAERALFLWNNEPIENLIIQNRNVILPIILPALEKNSRNHWNQAVRSLTLNVRKIFDDLDPELSNECLHKFEEAERKEDEIKARCEAKWERLEELAAQKALSSKAC*

>HbPP2AB'θ-2

MLKQILSKLPRKSFKSSQNRDHGGNLAINSNNSTGSRSSDFGEKSSKLNVTSVTANNSASDTGQNHGNNDQRGVTSKLNDNLGFSSYEGLPELRDVPNSEKQNLFIKKLNLCCIVFDFNDPTKNLKEKEIKRQTLLELVDYVTSVNGKFTETVIQEVLKMVSANLFRMFTPQPRENKVVDGVDLEEEEPSMDLAWPHLQLVYEVFLRFVVSSETDVKLAKRCIDQFFILKFLDLFDSEDPREREYLKTILHRIYGKFMVHRPFIRKAINNIFYQFIFETEKHNGMAELLEILGSIINGFALPLKEEHKMFLIHVLIPLHKPRCLAMYHQPLSYCIMQFVEKDYNLSDAIIRGLLKYWPLTNSSKEVMFLNELEEVLEATRPAEFQRCMVPLFLQIARCLNSSHFQVAERALFLWNNDAIESLIIQNRKVILPIIFPALEKNTRNHWNQAVRSLTLNVRKIFYDLEPELSNECLLNFEEAERKEDENKARCEATWKRLEELASQKT*

>HbPP2AB'κ-1

MLKQILSKLPRKSLKPESADLTSPESQSTQRSNGSSTGSRAKSNGPKRTSSAVFPASVVAGIEPLVPFKDVSSAERMNLFVRKVSLCCVTFDFTDPTKNTLEKDVKRQTLLELVDFVASGSMKFSEPAILAMCRMSAVNLFRVFPPNYRSNSISMSENDDNDEPVFDPAWPHLQIVYDLLLRFITSTCLDAKVAKKYIDHSFILRLLDLFDSEDPRERDCLKTILHKVYGKFMVHRPFIRKSISNIFYRFVFETEKHNGIAELLEIFGSIISGFALPLKEEHKIFLWRVLIPLHKPKSLGIYFQQLSYCVTQFMEKEPKLASIVIKGILKYWPITNSQKELMFLGELEEILEAINMVEFQKVMVPLFWRIGCCISSFHFQVAERALFLWNNDQIVNLIAHNRHAILPIIFPALEKNAHSHWNQAVLNLTLNVRKLFSEMDEALFLSCLAQFKDSEEKLSLLAEKRKVAWQQLENAASLKPITGNTAVLVTPFATSIIC*

>HbPP2AB'κ-2

MSISAENCCIATMLKQILSKIPRKSSKSDSFDSTGIDSGNNSNNWDSGVSCTNGGHSFSSRLNVVKRVSSAVFPASIMAGVEAVEPHISFKEVSNLQKQNLFVSKLNFCCKVFDFNDPDKIAAQQDIKHQTLMELVDFVSSGSAKFNEAAMAAMCKMCAINLFRVFPPKYLSNNTGGETDIEEPMFDPYWSHLQCVYDLLLRFISSVDAKAAKKHIDHAFIVRLLDLFDSEDPRERDCLKTILHRIYGNFMVHRPFIRKAVSNIIYHFVFETDHHNGIAELLEIFGSVISGFALPLKEEHKLFLWRALIPLHKPKSVGVYHQQLTYCVVQFIDKDPKLASTVIRGLLKYWPVTNSQKELMFISEIEEILEMTTMEEFQKIMVPLFRRMGCCLNSSHYQVAERAHLLWNNERILNLIVHNRQVIVPLVFSALERNAQNHWNQAVLNLTQNIRKMFCEMDEELVLACRSKLEEEDSMLSLKAEKRRLTWERLENAAGFQSAADNILAPVKPATCSVAC*

>HbPP2AB'μ

MNAHRNSAKASPGRISTTLKHLFDLDSKNNSNKLSSSPKIVRQSSSDPESEGILSLISYCSVIYTFTDPLESPSQQDIKRNKLIQLLSIVKSPKMSLPEQTLSPLVNMISANLFQPLPPSNNNAITADLLEDEEFASALSPTWPHLQLVYDILLRLVLNVDPKVLSNYIDQSFLTNVLCLFQSEDPRERESLKNVYHRIYSRFTFYRSFMRKSMNEVFLHYIFETEKHCGIGELLEIWGSIINGFTVPLKEEHKLFLMRVLIPLHKTKGMQVYHLQLSYCVNQFVQKEPMLGGIVVRGVLRYWPITNCHKEVLLIGELEELVDNIDPDHYRKLALPLCTQITRCLNSWNSQVAERALYIWNNEQFVKMANSAIEEVFPVVVEGMEKNLKWHWSQSVKQLTENVKAMLEEMDPSLYEKCLQEIGHKEYLAQQEEVKRKKRWERIELEAAKSQFLQPQKFICVSH*

>HbPP2AB''α

MDIDMAGGGGDAPYLDPELLQLPEVSPFVLKASPQIAEELFSQWLSLPGTSRLVKSLIDDAKIGNPVNFFGISVNTSAGGGNSLPSMFPAGSAPPLSPRSSSGSPRMSKQKTSPSSLGSPLKLVSEPVREVIPQFYFRNGRPPADELKEQCLSRINQFFSNHLDGLRIDEFKSITKQICKLPSFFSTALFRKIDSDCSGIVTRDAFIKYWVDGNMLAMDRTTCIFNILKQPENKYLIQTDFKPVLRELLAMHPGLEFLQSTPEFQERYAETVIYRIFYYINRSGNGRLTLRELKRGNLVAAMQHADEEEDINKVLRYFSYEHFYVIYCKFWELDTDHDFLIDKENLIRYGNHALTYRIVDRIFSQVPRKFTSNIEGKMGYEDFVYFMLSEEDKSSEPSLEYWFKCIDLDGNGVLTPNEMQFFYEEQLHRMECMAQEPVLFEDILCQIVDMIAPEANT*

>HbPP2AB''β

MDIDMAGGGGDAPYLDPELLQLPEVSPFVLKASPQIAEELFSQWLSLPGTSRLVKSLIDDAKIGNPINFFGISVNTSAGGGNSLPSMFPAGSAPPLSPRSSSGSPRMSKQKTSPSSLGSPLKLVSEPVREVIPQFYFRNGRPPANELKEQCLSRINQFFSNHLDGLRIDEFKSITKQICKLPSFFSTALFRKIDSDCSGIVTRDAFIKYWVDGNMLAMDRTTCIFNILKQPENKYLIQTDFKTVLRELLATHPGLEFLQSTPEFQERYAETVIYRIFYYINRSGNGRLTLRELKRGNLVAAMQHADEEEDINKVLRYFSYEHFYVIYCKFWELDTNHDFLIDKENLIRYGNHALTYRIVDRIFSQVPRKFTSNIEGKMGYEDFVYFMLSEEDKSSEPSLEYWFKCIDLDGNGVLTPNEMQFFYEEQLHRMECMAQEPVLFEDILCQIVDMIAPEVEDYITLHDLKTSKLSGNVFNILFNLNKFMAFESRDPFLIRQEHEEPTLTEWDRFAHREYIRLSMEEDVEDVSNGSADVWDESLEAPF*

>HbPP2AB''δ

MDTSPVAVGDVAMLDAELLQLPEVAPLAFKSYPDFAQKLFEQWLALPDANRLVTSLLNDAKAGAPLNVSGNSSNTTATTSSSLPSMFPGGSTPPLSPRSTSGSPRITKQRAGPSNLGSPLKVVSEPVKELIPQFYFLNGRPPPNELREQCLLRINHFFYGRLDGLQLHEFKSVTKEICKLPSFFSTVLFRRIDVNGTGFVTRDAFVHYWLNGNMLMMDMATQMFKILKQPDLKYLTQEDFKPVLRELLATHPGLEFLQSTPEFQERYAETVIYRIFYYINRSGNGHLTLRELKRGNLIDAMQHADEEEDINKVLRYFSYEHFYVIYCKFWELDTDHDFLIDKENLIRYGNHALTYRIVDRIFSQVPRKFTSRVEGKMGYEDFVYFILSEEDKSSEPSLEYWFKCIDLDGNGVLTRNEMQFFYEEQLHRMECMAQEPVLFEDILCQIIDMIGPENESYITLHDLKGCKLSGSVFNILFNLNKFMAFETRDPFLIRQERENPTLTEWDRFAHREYIRLSMEEDVEDASNGSAEVWDESLEAPF*

>HbPP2AB''ε

MDTVGDVAMLDAELLQLPEVAPLAFKSYPDFAQKLFEQWLALPDANRLVTSLLNDAKAGAPLNVSGNSSNATATTSSSLPSMFPGGSTPPLSPRSTSGSPRIMKQRAGPSNLGSPLKVVSEPVKELIPQFYFQNGRPAPNELKEQCLLRINHFFYGRSDGLQLHEFKSVTKEICKLPSFFSTVLFRRIDVNGTGFVTRDAFVNCWLNGSMLTMDTATQMFKILKQPDLKYLTQEDFKPVLRELLATHPGLEFLQSTPEFQERYAETVIYRIFHYINRSGNGHLTLRELKRGNLIDAMQHADEEEDINKVLRYFSYEHFYVIYCKFWELDMDHDFLIDKENLIRYGNHALTYRIVDRIFSQVPRKFTSKVEGKMGYEDFVYFILSEEDKSSEPSLEYWFKCIDLDGNGVLTRNEMQFFYEEQLHRMECMAQEPVLFEDILCQIIDMIGPENESYITLSDLKGCKLSGSVFNILFNLNKFMAFETRDPFLIRQERENPTLTEWDRFAHREYIRLSMEEDVEDASNGSAEVWDESLEAPF*

>HbPP2A-TON2/FASS1-1

MYSGSSDGESHEAAQRKIPPASSMPWVRNLRRYIGSGAGLGSEALMELETKRILLDIFKEKQQKSAEAGTIPSFYKKKPEEGSISHRVQRLAKYRFLNKQSDLLLNADDLDAMWVCLRENCVIDDATGAEKMNYEDFCHIASVCTEQIGPKCRRFFSPSNFMKFEKDESGRIAILPFYLYVMRTVSLTQARIDMSELDEDSDGFLQPHEMEAYIRGLIPNLAQLRDMPAQFVQMYCRIAAHKFFFFCDPHRRGKACIKKVLLSNCLQELMELHQESEEEVTDTEQAENWFSLTSAQRICDMFLALDKDMNGTLSKQELREYAEGTLTEIFIERVFDEHVRRGKSGGGNAREMDFESFLDFVLALENKDTPEGLTYLFRCLDLHGRGYLTTADIHSLFRDVHQKWIEGGNYELCIEDVRDEIWDMVKPTDPLKITLSDLLSCKQGGTVASMLIDVRGFWAHDNRENLLQEEEEPEEE*

>HbPP2A-TON2/FASS1-2

MYCGSIDGESHEASQRKIPPASSMLWVRNLRQYIASGAGLGSEALMELETKRILLDIFKEKQQNSAEAGTIPSFYKKKPEEGSISRRVQRLAKYRFLKKQSDLLLNADDLDAMLVCLRENCVIDDATGAEKMNYEDFCHIASVCTEQIGPKCRRFFSPSNFMKFEKDESGRIAILPFYLYVMRTVSLTQARIDMSELDEDSDGFLQPHEMEAYIQGLIPNLAQLRDMPAQFVQMYCRIAAHKFFFFCDPHRRGKACIKKVLLSNCLQELMELHQESEEEVTDTEQAENWFSLTSAQRICDMFLALDKDMNGTLSKQELREYADGTLTEIFIERVFDEHVRRGKSGGGHAREMDFESFLDFLLALENKDTPEGLTYLFRCLDLHGRGYLTTADIHSLFRDVHQKWIEGGNYELCIEDVRDEIWDMVKPTDSLRITLTDLLSCKQGGTVASMLIDVRGFWAHDNRENLLQEEEEPGEE*

>HbPP2AB55α/Bα-1

MNGAGSAEAAAVASVGVPLQPLEWKFSQVFGERTAGEEVQEVDIISAIEFDRTGNYLATGDRGGRVVLFERTDTREHDGQRRDLERMDISINRHPEFRYKTEFQSHEPEFDYLRSLEIEEKINKVRWCQSANGALFLLSTNDKTIKFWKVQEKKVKKVCDMNVDPENATGNGPILGSSMPTSSNCKQYTANGGSLDGPFGFPSNDFSFSPGGVPNLHLPMLTCHETSIVARCRRTYPHAHDYHINSISNNSDGETFISADDLRINLWNLEICNQSFNIVDVKPANMEDLTEVITSAEFHPTHCNMLAYSSSKGSIRLIDMRQSALCDSHSKLFEEKEVPGSKSFFTEIIASISDIKFAKDGRHILSRDYMNLKLWDINMDSGPVASFQVHEYLRPKLCDLYENDSIFDKFECCLSGDGLRMATGSYSNLFRVFGCSEGSTEASTLEATKNPMRRQVQTLLSGSSRSLGTLSRGFRRGADNTGVNTNGNAFDFTAKLLHLAWHPSDNLLACAASNSLYMYYA*

>HbPP2AB55α/Bα-2

MAYDVFLSSHFILLWSFAVDIISAIEFDRTGNHLATGDRGGRVVLFERTDTRVNDGNRRDLERMDFSINGHPEFRYKTEFQSHEPEFDYLRSLEIEEKINKIRWCQSANGALFLLSTNDKTIKFWKVQEKKVKKVCDMNVDPENAAGNGPILGSSLPTSSNSKPYTANGGSFDRPFGSPSNDFSFSPGGVPKLHLPVVALTSHETSVVARCRRIYAHAHDYHINSISNNSDGETFISADDLRINLWNLEISNQSFNIVDVKPANMEDLTEVITSAEFHPTHCNMLAYSSSKGAIRLIDMRQSALCDTHSKLFEEQEVPGSRSFFTEIIASISDIKFAKDGRHILSRDYMNLKLWDINMDSGPVASFQVHEYLRPKLCDLYENDSIFDKFECCLSGDGLRAATGSYSNLYRVFGCSKGSVEATTLEATKNPMRYV*

>HbPP2AB55α/Bα-3

MNDAGSAEAAAVASVGAPQPLEWKFAQVFGERAAGEEVQEVDIISAIEFDRTGNHLATGDRGGRVVLFERTDTRVNDGNRRDLERMDFSINGHPEFRYKTEFQSHEPEFDYLRSLEIEEKINKIRWCQSANGALFLLSTNDKTIKFWKVQEKKVKKVCDMNVDPENAAGNGPILGSSLPTSSNSKPYTANGGSFDRPFGSPSNDFSFSPGGVPKLHLPVLTSHETSVVARCRRIYAHAHDYHINSISNNSDGETFISADDLRINLWNLEISNQSFNIVDVKPANMEDLTEVITSAEFHPTHCNMLAYSSSKGAIRLIDMRQSALCDTHSKLFEEQEVPGSRSFFTEIIASISDVKFAKDGRHILSRDYMNLKVLFFIFVINFSLYVLCLKL*

>HbPP2AB55β/Bβ

MNGGDEVAAAPVGPPQPLEWKFSQVFGERTAGEEVQEVDIISAIEFDKTGDHLATGDRGGRVVLFERTDTKDHGGYRRDLERMDCPISRHPEFRYKTEFQSHEPEFDYLKSLEIEEKINKIRWCQTANGALFLLSTNDKTIKFWKVQEKKVKKISDMNVDPSKAVGNGSVASSSNSNGTKSYLANGGWPEKSYGYPSNDFTFPPGGIPSLHLPVVVTSNETSLVARCRRVYAHAHDYHINSISNNSDGETFISADDLRINLWNLEISNQSFNIVDVKPTNMEDLTEVITSAEFHPNHCNMLAYSSSRGSIRLIDLRQSALCDSHAKLFEEQEAPGSRSFFTEIIASISDIKFAKDGRHILSRDYMTLKLWDINMDSGPVATFQVQEYLRPKLCDLYENDSIFDKFECCLSGDGLRVATGSYSNLFRVFGCAPGSAEATTLEASKNPMRRQVQTPSRPSRSLSSITRVVRRGTESPGVDANGNSFDFTTKLLHLAWHPTENSIACAAANSLYMYYA*

>HbPP2AC1-1

MPSHGDLDRQIEHLMQCKPLTEAEVKALCEQARAILVEEWNVQPVKCPVTVCGDIHGQFHDLVELFRIGGNAPDTNYLFMGDYVDRGYYSVETVTLLVALKVRYRDRITILRGNHESRQITQVYGFYDECLRKYGNANVWKYFTDLFDYLPLTALIESQIFCLHGGLSPSLDTLDNIRSLDRIQEVPHEGPMCDLLWSDPDDRCGWGISPRGAGYTFGQDISQQFNHTNGLTLISRAHQLVMEGYNWSQDKNVVTVFSAPNYCYRCGNMAAILEIGDNMEQNFLQFDPAPRQVEPDTTRRTPDYFL*

>HbPP2AC1-2

MMLSHGVLDRQIEHLMQCKPLAEGEVKALCEQARAILVEEWNVQPVKCPVTVCGDIHGQFYDLVELFRIGGNAPDTNYLFMGDYVDRGYYSVETVTLLVALKVRYRDRITILRGNHESRQITQVYGFYDECLRKYGNANVWKYFTDLFDYLPLTALIESQIFCLHGGLSPSLDALDNIRALDRIQEVPHEGPMCDLLWSDPDDRCGWGISPRGAGYTFGQDISQQFNHTNGLTLISRAHQLVMEGYNWSQEKNVVTVFSAPNYCYRCGNMAAILEIGENMEQNFLQFDPAPRQIEPDTTRRTPDYFL*

>HbPP2AC2-1

MPSHGDLDRQIEHLMECKPLPEAEVKTLCDQAKAILVEEWNVQPVKCPVTVCGDIHGQFYDLIELFRIGGNAPDTNYLFMGDYVDRGYYSVETVTLLVALKVRYRDRITILRGNHESRQITQVYGFYDECLRKYGNANVWKYFTDLFDYLPLTALIESQIFCLHGGLSPSLDTLDNIRALDRIQEVPHEGPMCDLLWSDPDDRCGWGISPRGAGYTFGQDIAAQFNHTNGLTLISRAHQLVMEGYNWCQEKNVVTVFSAPNYCYRCGNMAAILEIGENMDQNFLQFDPAPRQIEPDTTRKTPDYFL*

>HbPP2AC2-2

MPSQGDLDRQIEHLMECKSLPEAEVKALCDQARAILVEEWNVQPVKCPVTVCGDIHGQFYDLIELFRIGGNAPDTNYLFMGDYVDRGYYSVETVTLLVALKVRYRDRITILRGNHESRQITQVYGFYDECLRKYGNANVWKYFTDLFDYLPLTALIESQVFCLHGGLSPSLDTLDNIRALDRIQEVPHEGPMCDLLWSDPDDRCGWGISPRGAGYTFGQDIAAQFNHTNGLTLISRAHQLVMEGYNWCQEKNVVTVFSAPNYCYRCGNMAAILEIGENMDQNFLQFDPAPRQIEPDTTRKTPDYFL*

>HbPP2AC4-1

MSLDSVLSNAHGNLDEQINQLMQCKPLSEQEVRMLCEKAKEILMEESNVQPVKSPVTICGDIHGQFHDLAELFRIGGKCPDTNYLFMGDYVDRGYYSVETVTLLVALKVRYPQRITILRGNHESRQITQVYGFYDECLRKYGNANVWKIFTDLFDYFPLTALVESEIFCLHGGLSPSIETLDNIRNFDRVQEVPHEGPMCDLLWSDPDDRCGWGISPRGAGYTFGQDISEQFNHTNNLKLIARAHQLVMEGYNWGHEQKVVTIFSAPNYCYRCGNMASILEVDDCKGHTFIQFEPAPRRGEPDVTRRTPDYFL*

>HbPP2AC4-2

MSLDSVPANAHGNLDEQINQLMQCKPLSEQEVRMLCEKAKEILMEESNVQPVKSPVTICGDIHGQFHDLAELFRIGGKCPDTNYLFMGDYVDRGYYSVETVTLLVALKVRYPQRITILRGNHESRQITQVYGFYDECLRKYGNANVWKIFTDLFDYFPLTALVESEIFCLHGGLSPSIETLDNIRNFDRVQEVPHEGPMCDLLWSDPDDRCGWGISPRGAGYTFGQDISEQFNHTNNLKLIARAHQLVMEGYNWGHEQKVVTIFSAPNYCYRCGNMASILEVDDCKGHTFIQFEPAPRRGEPDVTRRTPDYFL*

>HbPP2AC4-3

MGSNSLSSDSITDLDEQISQLIKCKPISEPQVRALCDKAKEILMQESSVQPVKSPVTICGDIHGQFHDLAELFRIGGKCPDTNYLFMGDYVDRGYYSVETVTLLVALKVRYPQRITILRGNHESRQITQVYGFYDECLRKYGNANVWKIFTDLFDCFPLTALVESEIFCLHGGLSPSIETLDGIRNFDRVQEVPHEGAMCDLLWSDPDDRCGWGISPRGAGYTFGQDISEQFNHTNNLKLIARAHQLVMDGFNWAHEQKVVTIFSAPNYCYRCGNMASILEVDDYKAHTFIQFEPAPRRGEPDVTRRTPDYFL*

>HbPP2AC6

MGANSLSSDSISDLDEQISQLMQCKPLSEPQVRALCDKAKEILMQESNVQPVKSPVTICGDIHGQFHDLAELFRIGGKCPDTNYLFMGDYVDRGYYSVETVTLLVALKVRYPQRITILRGNHESRQITQVYGFYDECLRKYGNANVWKIFTDLFDYFPLTALVESEIFCLHGGLSPSIETLDSIRNFDRVQEVPHEGAMCDLLWSDPDDRCGWGISPRGAGYTFGQDISEQFNHTNNLKLIARAHQLVMDGFNWAHVFIILAVHECPSGLCFQDSKFSL*

>AtPP2AA1

MAMVDEPLYPIAVLIDELKNDDIQLRLNSIRRLSTIARALGEERTRKELIPFLSENSDDDDEVLLAMAEELGVFIPFVGGIEFAHVLLPPLESLCTVEETCVREKAVESLCKIGSQMKENDLVESFVPLVKRLAGGEWFAARVSACGIFHVAYQGCTDVLKTELRATYSQLCKDDMPMVRRAAASNLGKFATTVESTFLIAEIMTMFDDLTKDDQDSVRLLAVEGCAALGKLLEPQDCVARILPVIVNFSQDKSWRVRYMVANQLYELCEAVGPDCTRTDLVPAYVRLLRDNEAEVRIAAAGKVTKFCRLLNPELAIQHILPCVKELSSDSSQHVRSALASVIMGMAPILGKDSTIEHLLPIFLSLLKDEFPDVRLNIISKLDQVNQVIGIDLLSQSLLPAIVELAEDRHWRVRLAIIEYVPLLASQLGIGFFDDKLGALCMQWLQDKVYSIREAAANNLKRLAEEFGPEWAMQHLVPQVLDMVNNPHYLHRMMVLRAISLMAPVMGSEITCSKFLPVVVEASKDRVPNIKFNVAKLLQSLIPIVDQSVVDKTIRQCLVDLSEDPDVDVRYFANQALNSIDGSTAAQS

>AtPP2AA2

MSMIDEPLYPIAVLIDELKNDDIQLRLNSIRRLSTIARALGEERTRKELIPFLSENNDDDDEVLLAMAEELGVFIPYVGGVEYAHVLLPPLETLSTVEETCVREKAVESLCRVGSQMRESDLVDHFISLVKRLAAGEWFTARVSACGVFHIAYPSAPDMLKTELRSLYTQLCQDDMPMVRRAAATNLGKFAATVESAHLKTDVMSMFEDLTQDDQDSVRLLAVEGCAALGKLLEPQDCVQHILPVIVNFSQDKSWRVRYMVANQLYELCEAVGPEPTRTELVPAYVRLLRDNEAEVRIAAAGKVTKFCRILNPEIAIQHILPCVKELSSDSSQHVRSALASVIMGMAPVLGKDATIEHLLPIFLSLLKDEFPDVRLNIISKLDQVNQVIGIDLLSQSLLPAIVELAEDRHWRVRLAIIEYIPLLASQLGVGFFDDKLGALCMQWLQDKVHSIRDAAANNLKRLAEEFGPEWAMQHIVPQVLEMVNNPHYLYRMTILRAVSLLAPVMGSEITCSKLLPVVMTASKDRVPNIKFNVAKVLQSLIPIVDQSVVEKTIRPGLVELSEDPDVDVRFFANQALQSIDNVMMSS

>AtPP2AA3

MSMVDEPLYPIAVLIDELKNDDIQRRLNSIKRLSIIARALGEERTRKELIPFLSENNDDDDEVLLAMAEELGGFILYVGGVEYAYVLLPPLETLSTVEETCVREKAVDSLCRIGAQMRESDLVEHFTPLAKRLSAGEWFTARVSACGIFHIAYPSAPDVLKTELRSIYGQLCQDDMPMVRRAAATNLGKFAATIESAHLKTDIMSMFEDLTQDDQDSVRLLAVEGCAALGKLLEPQDCVAHILPVIVNFSQDKSWRVRYMVANQLYELCEAVGPEPTRTDLVPAYARLLCDNEAEVRIAAAGKVTKFCRILNPELAIQHILPCVKELSSDSSQHVRSALASVIMGMAPVLGKDATIEHLLPIFLSLLKDEFPDVRLNIISKLDQVNQVIGIDLLSQSLLPAIVELAEDRHWRVRLAIIEYIPLLASQLGVGFFDEKLGALCMQWLQDKVHSIREAAANNLKRLAEEFGPEWAMQHIVPQVLEMINNPHYLYRMTILRAVSLLAPVMGSEITCSKLLPAVITASKDRVPNIKFNVAKMMQSLIPIVDQAVVENMIRPCLVELSEDPDVDVRYFANQALQSIDNVMMSS

>AtPP2AC1

MPLNGDLDRQIEQLMECKPLGEADVKILCDQAKAILVEEYNVQPVKCPVTVCGDIHGQFYDLIELFRIGGNAPDTNYLFMGDYVDRGYYSVETVSLLVALKVRYRDRLTILRGNHESRQITQVYGFYDECLRKYGNANVWKYFTDLFDYLPLTALIESQVFCLHGGLSPSLDTLDNIRSLDRIQEVPHEGPMCDLLWSDPDDRCGWGISPRGAGYTFGQDIATQFNHNNGLSLISRAHQLVMEGYNWCQEKNVVTVFSAPNYCYRCGNMAAILEIGEKMEQNFLQFDPAPRQVEPDTTRKTPDYFL

>AtPP2AC2

MPSNGDLDRQIEQLMECKPLSEADVRTLCDQARAILVEEYNVQPVKCPVTVCGDIHGQFYDLIELFRIGGNAPDTNYLFMGDYVDRGYYSVETVSLLVALKVRYRDRLTILRGNHESRQITQVYGFYDECLRKYGNANVWKYFTDLFDYLPLTALIESQVFCLHGGLSPSLDTLDNIRSLDRIQEVPHEGPMCDLLWSDPDDRCGWGISPRGAGYTFGQDIAAQFNHNNGLSLISRAHQLVMEGFNWCQDKNVVTVFSAPNYCYRCGNMAAILEIGENMEQNFLQFDPAPRQVEPDTTRKTPDYFL

>AtPP2AC3

MGANSIPTDATIDLDEQISQLMQCKPLSEQQCPDTNYLFMGDYVDRGYYSVETVTLLVALKMRYPQRITILRGNHESRQITQVYGFYDECLRKYGNANVWKIFTDLFDYFPLTALVESEIFCLHGGLSPSIETLDNIRNFDRVQEVPHEGPMCDLLWSDPDDRCGWGISPRGAGYTFGQDISEQFNHTNNLKLIARAHQLVMDGYNWAHEQKVVTIFSAPNYCYRCGNMASILEVDDCRNHTFIQFEPAPRRGEPDVTRRTPDYFL

>AtPP2AC4

MGANSLPTDATLDLDEQISQLMQCKPLSEQQVRALCEKAKEILMDESNVQPVKSPVTICGDIHGQFHDLAELFRIGGKCPDTNYLFMGDYVDRGYYSVETVTLLVGLKVRYPQRITILRGNHESRQITQVYGFYDECLRKYGNANVWKIFTDLFDYFPLTALVESEIFCLHGGLSPSIETLDNIRNFDRVQEVPHEGPMCDLLWSDPDDRCGWGISPRGAGYTFGQDISEQFNHTNNLKLIARAHQLVMDGFNWAHEQKVVTIFSAPNYCYRCGNMASILEVDDCRNHTFIQFEPAPRRGEPDVTRRTPDYFL

>AtPP2AC5

MPPATGDIDRQIEQLMECKALSETEVKMLCEHAKTILVEEYNVQPVKCPVTVCGDIHGQFYDLIELFRIGGSSPDTNYLFMGDYVDRGYYSVETVSLLVALKVRYRDRLTILRGNHESRQITQVYGFYDECLRKYGNANVWKHFTDLFDYLPLTALIESQVFCLHGGLSPSLDTLDNIRSLDRIQEVPHEGPMCDLLWSDPDDRCGWGISPRGAGYTFGQDIATQFNHTNGLSLISRAHQLVMEGFNWCQEKNVVTVFSAPNYCYRCGNMAAILEIGENMDQNFLQFDPAPRQVEPETTRKTPDYFL

>AtPP2AB55α/Bα

MNGGDEVVAASADPSLPLEWRFSQVFGERSAGEEVQEVDIISAIEFDNSGNHLATGDRGGRVVLFERTDTNNSSGTRRELEEADYPLRHPEFRYKTEFQSHDPEFDYLKSLEIEEKINKIRWCQTANGALFLLSTNDKTIKFWKVQDKKIKKICDMNSDPSRTVGNGTVASSSNSNITNSCLVNGGVSEVNNSLCNDFSLPAGGISSLRLPVVVTSHESSPVARCRRVYAHAHDYHINSISNNSDGETFISADDLRINLWNLEISNQSFNIVDVKPAKMEDLSEVITSAEFHPTHCNMLAYSSSKGSIRLIDLRQSALCDSHSKLFEEPEQAGPKSFFTEIIASVSDIKFAKEGRYLLSRDYMTLKLWDINMDAGPVATFQVHEYLKPKLCDLYENDSIFDKFECCISGNGLRAATGSYSNLFRVFGVAPGSTETATLEASRNPMRRHVPIPSRPSRALSSITRVVSRGSESPGVDGNTNALDYTTKLLHLAWHPNENSIACAAANSLYMYYA

>AtPP2AB55β/Bβ

MNGGDDAATSGPPPSLEWRFSQVFGERTAGEEVQEVDIISAIEFDKSGDHLATGDRGGRVVLFERTDTKDHGGSRKDLEQTDYPVRHPEFRYKTEFQSHEPEFDYLKSLEIEEKINKIRWCQPANGALFLLSTNDKTIKYWKVQEKKIKKISEMNIDPSESSNIPPQLVTNGLPADKGHDYLSKDFSFPPGGIPSLRLPVVTSQETNLVARCRRVYAHAHDYHINSISNSSDGETFISADDLRVNLWNLEISNQSFNIVDVKPTNMEDLTEVITSAEFHPIHCNMLAYSSSKGSIRLIDMRQSALCDSHTKLFEEPEAPGSRSFFTEIIASISDIKFSKDGRYILSRDYMTLKLWDINMDSGPVASYQVHEHLRPRLCDLYENDSIFDKFECCLSGDGLRVATGSYSNLFRVFGASQGSTEAATLEASKNPMRRQIQTPARPSRSIGSMTRVVRRGSESPGTEANGNAYDFTTKLLHMAWHPTENSIACAAANSLYMYYA

>AtPP2AB'α

MFKKIMKGANRKASKAEANDSSMYGFDPPGRSGPGSNMIVNHASRGSLVPSSPNSMAAATTQPPPMYSVEPLPLFRDVSVSERQSLFLRKLQICCFQFDFTDTLKNAREKEIKRQTLLELVDFIQSGAGKLTEVCQEEMVKMISVNIFRCLPPASHENTGQEPADLEEEEPYLEPSWPHLQLIYELLLRYIVPSDTDTKVAKRYIDHSFVLRLLELFETEDPREREYLKTILHRIYGKFMVHRPFIRKAMNHIFYRFIYETERHSGIGELLEILGSIINGFALPMKEEHKLFLIRALIPLHKPKPIAMYHQQLSYCIVQFVEKDYKLADTVIRGLLKFWPVTNCTKEVLFLGELEEVLEATQTVEFQRCMVPLFQQIARCLSSSNFQVAERALFLWNNEHVVGLIAQNRGVILPIIFASLEKNIESHWNQAVHGLSANIKRMFMEMDPELFEECQQQYEEKQAKSKQVEEQRQNRWRRLDEAVEEREREDPMITS

>AtPP2AB'β

MFKKIMKGGHRKPSKSEANEPSSYGIGLPDNRSGPGSNVVVSHASRGALVNSSPSPVTATPPPPPLGSVEPLPLFRDVPVSERQTLFLRKLQNCCFLFDFTDTIKNARDKEIKRQTLLELVDFIQSGSSKISESCQEEMIKMISVNIFRSLPPASHENTGQEPADPEEEEPYLEPSWPHLQLVYELLLRYVVSTDTDTKVAKRYIDHSFVLKLLDLFDSEDPREREYLKTILHRIYGKFMVHRPFIRKAINNIFYRFIYETERHSGIGELLEILGSIINGFALPMKEEHKLFLIRVLIPLHKPKPIVVYHQQLSYCIVQFVEKDYKLADTVIRGLLKYWPVTNCSKENLFLGELEEVLEATQPVEFQRCMVPLFQQIGRCLTSSHFQVAERALFLWNNEHIVGLIAQNRSVILPIIYPTLEKNIQSHWNQAVHGLTTNIKKMFMEMDPELFEECQRQYEEKQAKSKEVEEQRQYTWKRLAEAAAERDGGGGEEDHMITS

>AtPP2AB′γ

MIKQIFGKLPRKPSKSSHNDSNPNGEGGVNSYYIPNSGISSISKPSSKSSASNSNGANGTVIAPSSTSSNRTNQVNGVYEALPSFRDVPTSEKPNLFIKKLSMCCVVFDFNDPSKNLREKEIKRQTLLELVDYIATVSTKLSDAAMQEIAKVAVVNLFRTFPSANHESKILETLDVDDEEPALEPAWPHLQVVYELLLRFVASPMTDAKLAKRYIDHSFVLKLLDLFDSEDQREREYLKTILHRIYGKFMVHRPFIRKAINNIFYRFIFETEKHNGIAELLEILGSIINGFALPLKEEHKLFLIRALIPLHRPKCASAYHQQLSYCIVQFVEKDFKLADTVIRGLLKYWPVTNSSKEVMFLGELEEVLEATQAAEFQRCMVPLFRQIARCLNSSHFQVAERALFLWNNDHIRNLITQNHKVIMPIVFPAMERNTRGHWNQAVQSLTLNVRKVMAETDQILFDECLAKFQEDEANETEVVAKREATWKLLEELAASKSVSNEAVLVPRFSSSVTLATGKTSGS

>AtPP2AB′δ

MFKQILGKLPKKTSAKFWDNGESQTLDNNNNQGGGDEVLSQRTSSNGDTSLDCVSSFDVLPRLRDVSISEKQELFLKKLRLCCLVFDFVAEPQQNFKEKEIKRQTLLEVVDYVISSGNGKFPESVIQEATKMISANLFSNPHRQWKNKTPEALDLEEEEGSLNPSWPHLQIVYEFLLRIVASPNTDPKISKKYIDHTFVLKLLDLFDSEDPREREYLKTILHRIYGRFMVHRPFIRKTMNNILYDFIFETGKHSGIAEFLEVLGSIINGFALPLKEEHKLFLTRVLIPLHKLKCLPNYHQQLSYCVIQFVEKDCKLADTVIRGMLKYWPVTNSAKEIMFLNELEEILEATQLTEFERCMVPLSRQIAQCLSSSHFQVAERALYLWNNDHVTNLVRQNSRIILPIVFPALEKNGSSHWNQAVKNLTENVLKVLSDTNPDLFEECLHKFQEDQQKAEDTKKKNGETWRQLEEIVASMAK

>AtPP2AB′ε

MFNKIIKLGQKKFNKSDQHHQDNNNNNNNTSTNTVVRGSRTTTPAPSSVSNGESQTTAQSPSQTPNHPMFTTTPILEVLPLLKDVSSSDRPLLFMKKAHMCSCHCDFSDTLIMPREKEIKRQTLLELVDFLHSSSGKVNETMQSELIRMVSANIFRCLPPAYHENTGAPPEGNDPEEEEPYLEPWWPHLQLVYELLLRYVVSSEIEPKTAKKFINHTFVSRLLDLFDSEDPREREYLKTVLHRIYGKFIFHRPFIRCSIYNIFYKFLYETERCIGIGELLEILGSVINGFTVPMREEHRLYLVKAILPLHKSKGISIYHQQLAYCVTQFVEKDYKLADTVIRGLLKFWPLTNCQKEVLFLGELEEVLDATEPSEFQQCVVPLFTQIGKCLNSAHFQVAERALFLWNNEHIVGLIAQNKDVIFPIIFEALERNMKGHWNQAVHGLSENVRRMFLEMDTELFEECEKQYLENEAKACELLEQRELTWKRLEEAASLAAN

>AtPP2AB′ζ

MIKQIFGKLPRKPSKSLQNDSNGEGGVNNSYYASNSSTTSISKPSSTSSKSSSASGSRVANGTLAPNSMSSNRNTNQGKKPLGGDAVVQAGPFPSSGGVYEALPSFRDVPISEKPNLFIGKLSMCCVVFDFSDPSKNLKEKEIKRQTLLELVDYVASVGFKFNDVSMQELTKMVAVNLFRTFPSANHESKILEIHDMDDEEPSLEPAWPHVQVVYEILLRFVASPMTDAKLAKRYIDHSFVLKLLDLFDSEDQREREYLKTILHRVYGKFMVHRPYIRKAINNIFYRFISETEKHNGIAELLEILGSIINGFALPLKEEHKLFLLRALIPLHKPKCSSVYHQQLSYCIVQFVEKDFKLADTVIRGLLKYWPVTNSSKEVMFLGELEEVLEATQAAEFQRCMVPLSRQIARCLNSSHFQVAERALFLWNNDHIRNLITQNHKVIMPIVFPALERNTRGHWNQAVQSLTINVRKVLCEIDQVLFDECLAKFQVEEVNKTEVKAKRERTWQRLEDLATSKTVVTNEAVLVPRFVSSVNLTTSSSESTGS

>AtPP2AB′η

MWKQILSKLPNKKSSKHEHRGREHGGHSSSSSHTSGASTSKSTDNGAAKSHAKNASPAGKSAASDSGFKDGNLKSSGNNNNNNNNGVFTPYEALPSFKDVPNTEKQNLFIKKLNLCRVVFDFTDPTKNIKEKDIKRQTLLELVDYVNSPNGKFSEVGIQEVVRMVSANIFRTLNPQPRENKVIDALDLEEEEPSMDLAWPHLQLVYELFLRFVASPETDTKLAKRYIDQSFVLRLLDLFDSEDPRERDCLKTILHRIYGKFMVHRPFIRKSINNIFYRFVFETEKHNGIAEFLEILGSIINGFALPLKDEHKVFLVRVLIPLHKPKCLQMYHQQLSYCITQFVEKDCKLADTVIRGLLKYWPVTNSSKEVMFLNELEEVLEATQPPEFQRCMVPLFRQIARCLNSLHFQVAERALFLWNNNHIENLIMQNRKVILPIIFPALERNAQKHWNQAVHSLTLNVRKIFHDLDPELFKECLAKFKEDESKAAETEAKREATWKRLEELGVRKAS

>AtPP2AB′θ

MWKQILSKLPKKSSSKNHSSSSSSTSKSSDNGASKSGNSQTQNAPPVKPSADSGFKEGNLKGNGNGFTPYEALPGFKDVPNAEKQNLFVRKLSLCCVVFDFSDPTKNVKEKDIKRQTLLELVDYVASPNGKFSETVIQEVVRMVSVNIFRTLNPQPRENKVIDALDLEEEEPSMDPTWPHLQLVYEILLRLIASPETDTKLAKKYIDQSFVSRLLDLFDSEDPRERDCLKTVLHRIYGKFMVHRPFIRKSINNIFYRFVFETEKHNGIAEFLEILGSIINGFALPLKDEHKVFLVRALVPLHKPKSLQMYHQQLSYCITQFVEKDCKLADTVIRGLLKSWPVTNSSKEVMFLNELEEVLEATQPPEFQRCMVPLFRQVARCLNSLHFQVAERALFLWNNDHIENLIMQNRKVILPIIFPALERNTQKHWNQAVHSLTLNVQKIFNDIDAELFKDCLAKFREDESKEAEIGAKREATWKRLEEIGNQKQKSSL

>AtPP2AB′κ

MFKQFLSKLPRKSSKSDSGELNRSSSGPVSSPVQRSGTSGGGSGPVRSNSGKRMSSAVFPASVVAGIEPLVPFKDVPSSEKLNLFVSKVSLCCVTFDFSDPGKNSIEKDVKRQTLLELLDFVASGSVKFTEPAILAMCRMCAVNLFRVFPPNYRSSSGGENDDDEPMFDPAWPHLQIVYDLLLKFITSPCLDAKVAKKYLDHAFIVRLLDLFDSEDPRERECLKTILHRVYGKFMVHRPFVRKSMSNIFYRFVFETEKHSGIAELLEIFGSIVSGFALPLKEEHKIFLWRVLIPLHKPKSVGNYFQQLSYCITQFIDKEPKLGSVVIKGLLKFWPITNSQKEVMFLGEVEEIVEAMSVMEFQKIMVPLFLRIACCVTSSHFQVSERALFLWNNDQIVNLIGHNRQAILPIMFTALEKNAQNHWNQSVLNLTLNVRKMFCEMDEALFMSCHARFKEDEAKQCSAAEKRKEVWARLENAASMKPITGKTAVLVTPRATSIAC

>AtPP2AB′′α

MEIDGGNDVQILDPELLQLPGLSPVSLKENPHIAEELFSQWLSLPETGRLVKSLIDDTKSSTPVSVSKNCTSLNVACGSALPSVFLNSGTPPLSPRGSPGSPRFSRQKTSPSLQSPLKSVREPKRQLIPQFYFQHGRPPAKELREQCISMVDQFFSNYIDGLHMDEFKSITKEVCKLPSFLSSVLFRKIDTSGTGIVTRDAFIKYWVDGHMLAMDVASQIYNILRQPGCKYLRQADFKPVLDELLTTHPGLEFLRNTPEFQERYAETVIYRIFYYINRSGTGCITLRELKRGNLITAMQQVDEEDDINKVIRYFSYEHFYVIYCRFWELDGDHDFLIDKENLIKYGNHALTYRIVDRIFSQVPRKFTSKVEGKMSYEDFAYFILAEEDKSSEPSLEYWFKCIDLDGDGVITPNEMQFFYEEQLHRMECITQEPVLFEDILCQIFDMIKPEKENCITLQDLKASKLSGNIFNILFNLNKFMAFETRDPFLIRQERENPTLTEWDRFAQREYVRLSMEEDVEEVSNGSADVWDEPLESPF

>AtPP2AB′′β

MVDQVFSNYIDGLHVDEFKSITKQVCKLPSFLSPALFRKIDPNCTDIVTRDAFIKYWIDGNMLTMDTASQIYNILRQQGCSYLRQADFKPVLDELLATHPGLEFLRTISEFQERYAETVIYRIFYYINRSGTGCLTLRELRRGNLIAAMQQLDEEDDINKIIRYFSYEHFYVIYCKFWELDGDHDCFIDKDNLIKYGNNALTYRIVDRIFSQIPRKFTSKVEGKMSYEDFVYFILAEEDKSSEPSLEYWFKCVDLDGNGVITSNEMQFFFEEQLHRMECITQEAVLFSDILCQIIDMIGPEKENCITLQDLKGSKLSANVFNILFNLNKFMAFETRDPFLIRQEREDPNLTEWDRFAQREYARLSMEEDVDEVSNGSADVWDEPLEPPF

>AtPP2AB′′γ

MESITLDIELLQLPETSPMSMKSNQDFVKKLFDQWLALPETNRLVTSLVNDAKAGVALNVMCGGGSSGTNSGSNSPLASMFPARNGPPLSPRNSTGSPRIARQRTGLSNLSSPLKVVSDHVKELIPQFYFEDGRPPPNDLKEQCIAKINSLFYGHEDGLQLQEFKLVTTEICKVPSFFSTSIFKKVDTNNTGFVKREDFIDYWVKGNMLTKEITSQVFTILKQPDHNYLVQDDFKPVLQELLATHPGLEFLQGTPEFQDRYAETVIYRIYYYINRSGNGHLTLRELKRGNLVDAMQHADEEEDINKVLRYFSYEHFYVIYCKFWELDTDHDFLIDKENLIRYSNHALTYRIVDRIFSQVPRKFTSKTEGKMGYEDFVYFILAEEDKSSEPSLEYWFKCIDLDANGVLTRNELQFFYEEQLHRMECMAQEAVLFEDILCQLFDMVKPEDEGFICLNDLKGSKLSGNVFNILFNLNKFMAFETRDPFLIRQERANPTWTEWDRFAHREYIRLSMEEDVEDASNGSAEAWDDSLEVPF

>AtPP2AB′′δ

MVDTVIPGDMACLDADLLQLQEMSSFVLNSKPGFTQKLFDQWLSLPEAQRQVGSLLKDAVAGAPINVTGSASGSNSATIPSMFPAGSAPPLSPRSCGSPRTTKQRAPSNLGSTLKVVNEPVKEPIPQFYFQNGRPPPSEIKEQCMFRINHFFYGHMDGLQIQEFKLVTREICKLPSFFSTSLFRKIDLNNTGFVTRDAFIDFWVNGNMLIMDTTTQIFKILKQKDQSFIVKDDFKPLLKELLATHPGLEFLQSTPEFQERYAETVTYRIFYYINRSGNGRITFRELKRGNLIDAMLHADEEEDINKVLRYFSYEHFYVIYCKFWELDTDHDFLIDKENLMRYGNHALTYRIVDRIFSQVARKFTNKVEGKMGYEDFVYFILAEEDKSSVPSLEYWFKCIDLDANGIITRNEMQFFYEEQLHRMECMAQEAVLFEDILCQMIDMIGPENESHITLHDLKGSKLSGNVFNILFNLNKFMAFETRDPFLIRQERENPTLTDWDRFAHREYIRLSMEEDVEDASNGSAEVWDESSLEAPF

>AtPP2AB′′ε

MVDTVIPGDMACLDADLLQLQEMSSFVLNSKPGFTQKLFDQWLSLPEAQRQVGSLLKDAVAGAPINVTGSASGSNSATIPSMFPAGSAPPLSPRSCGSPRTTKQRAPSNLGSTLKVVNEPVKEPIPQFYFQNGRPPPSEIKEQCMFRINHFFYGHMDGLQIQEFKLVTREICKLPSFFSTSLFRKIDLNNTGFVTRDAFIDFWVNGNMLIMDTTTQIFKILKQKDQSFIVKDDFKPLLKELLATHPGLEFLQSTPEFQERYAETVTYRIFYYINRSGNGRITFRELKRGNLIDAMLHADEEEDINKVLRYFSYEHFYVIYCKFWELDTDHDFLIDKENLMRYGNHALTYRIVDRIFSQVARKFTSKVEGKMGYEDFVYFILAEEDKSSVPSLEYWFKCIDLDANGIITRNEMQFFYEEQLHRMECMAQEAVLFEDILCQMIDMIGPENESHITLHDLKGSKLSGNVFNILFNLNKFMAFETRDPFLIRQERENPTLTDWDRFAHREYIRLSMEEDVEDASNGSAEVWDDSSLEAPF

>AtPP2A-TON2/FASS1

MYSGSSDGESHDTSTQRKIPPASSMLWVRNLRRYIGSGAGLGSEALMELETKRILLEIFKEKQQKSQEAGTIPSFYKKKPEEGSISQRVQKLAKYRFLKKQSDLLLNADDLAAMWVCLRENCVIDDATGAEKMNYEDFCHIASVCTEQIGPKCRRFFSPSNFMKFEKDEAGRIAILPFYLYVMRTVSLTQARIDMSELDEDSDGFLHSDEMESYIGGLIPNLAQLRDMPPAFNQMYCRIASQKFFFFCDPHRRGRACIKKILLSNCLQELMELHQESEEEVTDTEQAENWFSLTSAQRICDMFLALDKDMSGSLCKQELKEYADGTLTEIFIERVFDEHVRRGKIVAGNSREMDFDSFLDFVLALENKDTPEGLTYLFRCLDLQGRGFLTTADIHSLFRDVHQKWIEGGNYELCIEDVRDEIWDMVKPSDPLKITLGDLLGCKQGGTVASMLIDVRGFWAHDNRENLLQEEEEPPEEESQ
