# Supplementary material for: Genome-wide identification and expression analysis of the phosphatase 2A family in rubber tree (Hevea brasiliensis)
Source: PLoS One. 2020 Feb 5;15(2):e0228219. doi: 10.1371/journal.pone.0228219 (PMC7001923; doi:10.1371/journal.pone.0228219)
Supplement: S4 Table — (DOCX) [file pone.0228219.s005.docx]

**S4 Table. The Ka/Ks ratios and estimated absolute dates for the duplication events between the duplicated HbPP2As**

| Duplicated pair | Ka | Ks | Ka/Ks | Date (Mya) | Duplicate type | Purifying selection |
| --- | --- | --- | --- | --- | --- | --- |
| HbPP2AA1-1 vs HbPP2AA1-2 | 0.0298498 | 0.228253 | 0.130775 | 15.21687 | Segmental | Yes |
| HbPP2AB'η-1 vs HbPP2AB'η-2 | 0.0642869 | 0.160701 | 0.40004 | 10.7134 | Segmental | Yes |
| HbPP2AB'η-1 vs HbPP2AB'η-3 | 0.136663 | 1.23342 | 0.1108 | 82.228 | Segmental | Yes |
| HbPP2AB'η-1 vs HbPP2AB'η-4 | 0.130303 | 1.78212 | 0.0731167 | 118.808 | Segmental | Yes |
| HbPP2AB'η-1 vs HbPP2AB'η-5 | 0.13341 | 2.00908 | 0.0664037 | 133.9387 | Segmental | Yes |
| HbPP2AB'η-2 vs HbPP2AB'η-3 | 0.135506 | 1.29704 | 0.104473 | 86.46933 | Segmental | Yes |
| HbPP2AB'η-2 vs HbPP2AB'η-4 | 0.132366 | 1.45411 | 0.0910287 | 96.94067 | Segmental | Yes |
| HbPP2AB'η-2 vs HbPP2AB'η-5 | 0.149008 | 1.46799 | 0.101505 | 97.866 | Segmental | Yes |
| HbPP2AB'η-3 vs HbPP2AB'η-4 | 0.0375453 | 0.219227 | 0.171262 | 14.61513 | Segmental | Yes |
| HbPP2AB'η-3 vs HbPP2AB'η-5 | 0.0511121 | 0.266226 | 0.191988 | 17.7484 | Segmental | Yes |
| HbPP2AB'η-4 vs HbPP2AB'η-5 | 0.0171609 | 0.0379419 | 0.452295 | 2.52946 | Tandem | Yes |
| HbPP2AB'θ-1 vs HbPP2AB'θ-2 | 0.0555271 | 0.203847 | 0.272396 | 13.5898 | Segmental | Yes |
| HbPP2AB'κ-1 vs HbPP2AB'κ-2 | 0.240683 | 1.89683 | 0.126887 | 126.4553 | Segmental | Yes |
| HbPP2A-TON2/FASS1-1 vs HbPP2A-TON2/FASS1-2 | 0.016149 | 0.263228 | 0.0613496 | 17.54853 | Segmental | Yes |
| HbPP2AB55α/Bα-1 vsHbPP2AB55α/Bα-2 | 0.0530898 | 0.272405 | 0.194893 | 18.16033 | Segmental | Yes |
| HbPP2AB55α/Bα-1 vsHbPP2AB55α/Bα-3 | 0.0577099 | 0.235624 | 0.244924 | 15.70827 | Segmental | Yes |
| HbPP2AB55α/Bα-2 vsHbPP2AB55α/Bα-3 | 0.043209 | 0.132559 | 0.32596 | 8.837267 | Segmental | Yes |
| HbPP2AC1-1 vs HbPP2AC1-2 | 0.0149701 | 0.183535 | 0.0815654 | 12.23567 | Segmental | Yes |
| HbPP2AC2-1 vs HbPP2AC2-2 | 0.00718315 | 0.268933 | 0.0267098 | 17.92887 | Segmental | Yes |
| HbPP2AC4-1 vs HbPP2AC4-2 | 0.00274327 | 0.162139 | 0.0169193 | 10.80927 | Segmental | Yes |
| HbPP2AC4-1 vs HbPP2AC4-3 | 0.0488928 | 2.21246 | 0.0220988 | 147.4973 | Segmental | Yes |
| HbPP2AC4-2 vs HbPP2AC4-3 | 0.0477685 | 1.80787 | 0.0264224 | 120.5247 | Segmental | Yes |
