# Supplementary material for: Genome-wide identification and expression analysis of the phosphatase 2A family in rubber tree (Hevea brasiliensis)
Source: PLoS One. 2020 Feb 5;15(2):e0228219. doi: 10.1371/journal.pone.0228219 (PMC7001923; doi:10.1371/journal.pone.0228219)
Supplement: Attachment — Submitted filename: Response for MS.docx [file pone.0228219.s006.docx]

**Question for editor**:

1 Therefore, we invite you to submit a revised version of the manuscript that addresses all points raised during the review process, in particular toning down the conclusion that “moderately down-regulated expression of HbPP2As is crucial for the enhanced natural rubber biosynthesis in rubber trees”, as downregulation of PP2As at this point just correlates with enhanced rubber biosynthesis. To underscore a causal relationship, additional experiments are needed. In addition, qPCR data should be made available.

**Answer:** Thank you for your kind suggestion. In the new version: (1) we have rephrased the conclusions; (2) the qRT-PCR data is supplied as a attachment (S3 Table).

**Reviewer** 1:

1 Figure 6. For B55 Subfamily the gene names appear to be missing.

**Answer:** Thank you for your kind suggestion. We have added " HbPP2AB55α/Bα-1" and " HbPP2AB55β/Bβ" in the revised Fig 6 in new version.

**Reviewer** 2:

1 Have the authors made all data underlying the findings in their manuscript fully available?

**Answer:** Thank you for your kind suggestion. We have supplied the qRT-PCR data as attachment (S3 Table).

2 However, based on the evidence presented in the manuscript, I feel that the main conclusion “moderately down-regulated expression of HbPP2As is crucial for the enhanced natural rubber biosynthesis in rubber trees” is not fully justified. The study does not provide experimental evidence, that the moderate down regulation of PP2As is necessary for the enhanced rubber biosynthesis. Altogether, the manuscript presents interesting findings and new data about PP2As in *Hevea brasiliensis* but the authors should rephrase the conclusions of the manuscript.

**Answer:** Thank you for your kind suggestion. In the revised version, we suggest that down-regulated expression of HbPP2As may contribute to the enhanced phosphorylation of proteins by ethrel application. Whether the protein phosphorylized modification on the activity of rubber biosynthesi-related proteins remains elucidated.3 The labeling in the Figure 6 is misleading as the trees were treated with ethrel and not with ethylene.

**Answer:** Thanks. We have modified the labeling in the revised Fig 6 in new version.

4 Although the manuscript is written in good English, I feel that careful editing of the language would enhance the readability of the manuscript.

**Answer:** Thanks. We have re-checked the MS thoroughly in the revised version.

5 According to PlosOne policy, the qPCR data presented in the manuscript should be made available for the reader.

**Answer:** Thanks. We have supplied the qRT-PCR data as a attachment (S3 Table).
